# Zoledronic Acid for prevention of bone and muscle loss after BAriatric Surgery (ZABAS)-a study protocol for a randomized controlled trial

**DOI:** 10.1186/s13063-022-06766-z

**Published:** 2022-10-08

**Authors:** Søren Gam, Bibi Gram, Claus Bogh Juhl, Anne Pernille Hermann, Stinus Gadegaard Hansen

**Affiliations:** 1 Department of Medicine, University Hospital of Southern Denmark, Odense, Denmark; 2 Steno Diabetes Center, Odense, Denmark; 3 The Research Unit of Health Sciences, University Hospital of Southern Denmark, Odense, Denmark; 4 Department of Regional Health Research, University of Southern Denmark, Odense, Denmark; 5 Department of Endocrinology, Odense University Hospital, Odense, Denmark

**Keywords:** Bariatric surgery, Gastric bypass, Sleeve gastrectomy, Bone loss, Muscle loss, Zoledronic Acid, Bone microarchitecture, Volumetric bone density, Lean body mass, Muscle mechanical function

## Abstract

**Background:**

Bariatric surgery has adverse effects on the muscular-skeletal system with loss of bone mass and muscle mass and an increase in the risk of fracture. Zoledronic acid is widely used in osteoporosis and prevents bone loss and fracture. Bisphosphonates may also have positive effects on skeletal muscle. The aim of this study is to investigate the effects of zoledronic acid for the prevention of bone and muscle loss after bariatric surgery.

**Methods/design:**

This is a randomized double-blind placebo-controlled study. Sixty women and men with obesity aged 35 years or older will complete baseline assessments before randomization to either zoledronic acid (5 mg in 100 ml isotonic saline) or placebo (100 ml isotonic saline only) 3 weeks before surgery with Roux-en-Y-gastric bypass (RYGB) or sleeve gastrectomy (SG). Follow-up assessments are performed 12 and 24 months after surgery. The primary outcome is changes in lumbar spine volumetric bone mineral density (vBMD) assessed by quantitative computed tomography (QCT). Secondary bone outcomes are changes in proximal femur vBMD assessed by QCT. Changes in cortical and trabecular bone microarchitecture and estimated bone strength will be assessed by high-resolution peripheral quantitative computed tomography (HR-pQCT). Cortical material bone strength at the mid-tibia diaphysis will be assessed using microindentation and fasting blood samples will be obtained to assess biochemical markers of bone turnover and calcium metabolism.

Secondary muscle outcomes include whole body lean mass assessed using dual-energy X-ray absorptiometry. Dynamometers will be used to assess handgrip, shoulder, ankle, and knee muscle strength. Short Physical Performance Battery, 7.6-m walking tests, 2-min walking test, and a stair climb test will be assessed as biomarkers of physical function. Self-reported physical activity level is assessed using International Physical Activity Questionnaire (IPAQ).

**Discussion:**

Results from this study will be instrumental for the evidence-based care of patients undergoing bariatric surgery.

**Trial registration:**

ClinicalTrials.gov NCT04742010. Registered on 5 February 2021.

**Supplementary Information:**

The online version contains supplementary material available at 10.1186/s13063-022-06766-z.

## Background

Obesity, defined as a body mass index (BMI) > 30 kg/m^2^, affects an increasing number of individuals worldwide and is associated with cardiovascular disease, diabetes mellitus, and increased mortality [1]. During several decades, bariatric surgery has been increasingly used to treat people with severe obesity especially using the Roux-en-Y gastric bypass (RYGB) procedure, with sleeve gastrectomy (SG) becoming increasingly popular in recent years [2]. Bariatric surgery has, however, adverse effects in the muscular-skeletal system as reflected by decreasing bone and muscle mass [3–7].

The loss of weight after bariatric surgery is mainly achieved within the first 6–12 months post-surgery and typically, weight is stable or slightly increasing afterwards. Despite weight stability, the age-related decline in bone mineral density (BMD) is accelerated, bone turnover remains increased, and bone microarchitecture deteriorates which cause a reduction in estimated bone strength in the years following surgery [3, 6, 7]. This happens in parallel with an average of 25% decline in body lean mass 2 years after surgery and reduction in absolute muscle strength [4, 5]. Altogether, this may cause a higher risk of falls and a reduction in bone strength that may both contribute to an increase in fracture risk. Several studies have documented that people undergoing bariatric surgery have an increased risk of fracture [8–11] both compared to individuals with similar age, sex, and BMI and the general population and this risk ratio seems to increase further with time from surgery [10–12].

The negative skeletal effects of bariatric surgery are presumably multifactorial, and mechanisms may involve nutritional factors, mechanical unloading, hormonal factors, and changes in body composition and bone marrow fat [13]. After bariatric surgery and RYGB in particular, the gastrointestinal absorption of calcium and vitamin D is compromised which causes secondary hyperparathyroidism and an increase in bone remodeling [14]. Compromised absorption of nutrients and skeletal unloading, however, do not fully account for the increase in bone resorption, and changes in hormones related to adipose tissue and glucose homeostasis hormones may also be of importance. Furthermore, major changes are observed in the secretion of gastrointestinal hormones such as serum peptide YY, gastric inhibitory protein (GIP), and glucagon-like-peptide 1 and 2 (GLP1 and GLP2) that have been shown to modulate bone remodeling [13].

Following bariatric surgery patients are routinely advised supplements with calcium and vitamin D. Also, to preserve muscular-skeletal health after surgery, nutrition or exercise regimens or both in combination have been tested to prevent the loss of bone and of muscle mass and muscle strength after bariatric surgery [4, 5, 15], although with limited success. Therefore, interventions that can preserve muscular-skeletal health when undergoing bariatric surgery are warranted.

The bisphosphonate zoledronic acid is widely used for prevention of bone loss and fracture in women and men with osteoporosis [16, 17]. It acts through inhibition of osteoclasts. It is administered once yearly although effects in most cases last up to 2 to 3 years after administration [18]. The use of bisphosphonates in a setting of bariatric surgery is unclarified. Two ongoing randomized clinical trials (NCT03411902 and NCT0427939) that use bisphosphonates are also aiming to clarify this matter. Preliminary results in a sample size of four showed that zoledronic acid may preserve trabecular bone density in the spine 24 weeks post-surgery but was not able to prevent bone loss in the hip [19].

In addition to preserving bone mass, animal and small-scale human studies indicate that bisphosphonates may also be able to preserve muscle mass [20–22] and improve muscle strength and physical performance [23, 24]. Bisphosphonates may prevent muscle wasting by downregulating the osteoclast activity and thereby lower the release of cytokines like TGF-β that are embedded in the bone matrix. These cytokines have a catabolic effect on muscles [25]. Moreover, bisphosphonates are also reported to reduce pain and improve activities of daily living in postmenopausal women with existing vertebral fractures [26], which may lead to increased physical activity and subsequently preserve muscle mass.

In this randomized placebo-controlled study, we aim to assess the effects and safety of zoledronic acid on surrogate markers of bone strength in patients undergoing bariatric surgery. Also, effects on muscle mass and function will be assessed. We hypothesize that treatment with zoledronic acid will prevent the increase in bone turnover and preserve surrogates of bone strength in patients undergoing bariatric surgery. Additionally, we postulate that zoledronic acid will antagonize the post-surgery loss of muscle mass and function.

### Study objectives

To evaluate the effects of zoledronic acid on (1) bone mineral density, structure, material quality, and metabolism and (2) muscle mass, muscle strength, physical function, and physical activity in patients after bariatric surgery.

## Methods/design

### General design

This is a single-center randomized double-blinded, placebo-controlled study conducted at the University hospital of Southern Denmark. The study will be conducted in accordance with the Standard Protocol Items: Recommendations for Interventional Trials (SPIRIT) 2013 Checklist for RCTs (Additional file 1) [27] and the Declaration of Helsinki, approved by the Regional Committee on Health Research Ethics for Southern Denmark (project identifier S-20190134), Danish Medicine Agency (protocol number: Z0L6700), and registered at ClinicalTrials.gov (NCT04742010) and EudraCT (2019–001,650-26). In case protocol modifications are required during the trial, these will be reported to the relevant authorities. Enrolment began in February 2021 and is expected to be completed in approximately May 2022. The results will be reported in accordance with the CONSORT statement for trial reports and results, positive or negative, will be disseminated.

**Fig. 1:**
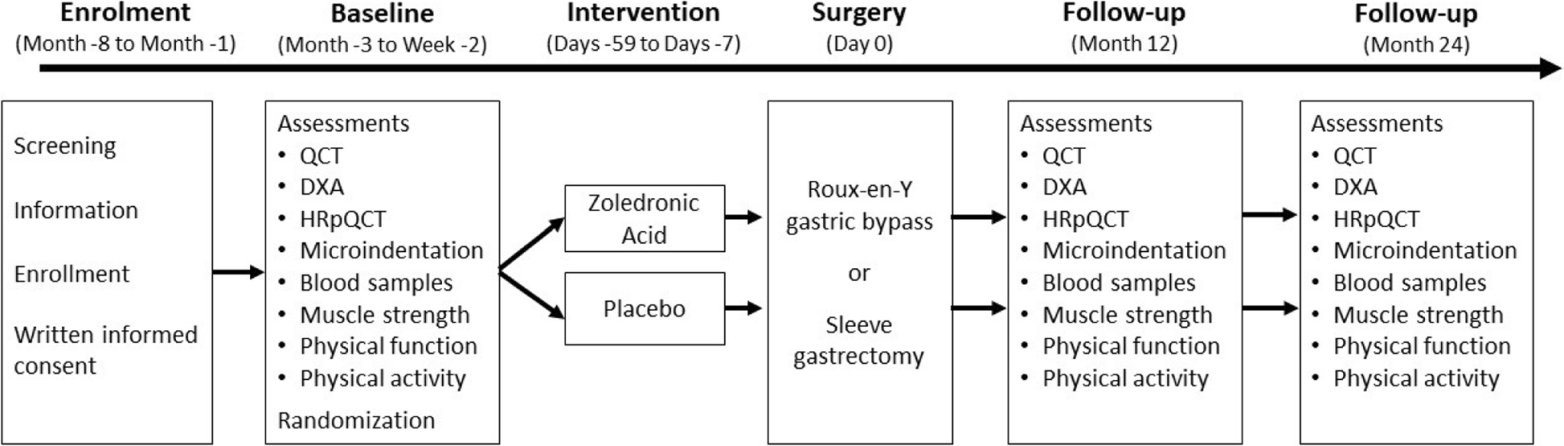
The study design. QCT, quantitative computed tomography; DXA, whole-body dual-energy X-ray absorptiometry; HR-pQCT, high-resolution peripheral quantitative computed tomography

Figure 1 shows the design of the study. After enrollment, the subjects complete baseline assessments before randomization to either zoledronic acid or placebo. The subject will receive zoledronic acid or placebo − 59 to − 7 days prior to surgery. Follow-up assessment will be performed 12 and 24 months after surgery. For the assessments at each time point, the subjects will visit the hospital on two occasions. At day 1, the following assessment will be conducted in the order: overnight fasting blood samples; quantitative computer tomography (QCT); dual-energy X-ray absorptiometry (DXA); muscle function; physical function; physical activity, and for day 2: high-resolution peripheral quantitative computed tomography (HR-pQCT); microindentation. Prior to day 1, the subject will be instructed to refrain from any strenuous physical activity and alcohol > 24 h before assessments.

### Subjects and recruitment

Patients referred for bariatric surgery (RYBG or SG) are invited to participate in the study. As part of their routine clinical visits, patients will be asked for potential interest in participating and if agreed written information will be mailed. A member of the study team will then contact the patients. After the oral information, the patient is given time to consider participation and afterwards the informed consent is obtained. The investigator was responsible for obtaining informed consent and enrolling patients in the study.

#### Inclusion criteria

Patients that are ≥ 35 years old and are eligible for bariatric surgery according to Danish National Guidelines will be included in the study. These include either (1) BMI ≥ 35 kg/m^2^ and an obesity-related comorbid condition of type 2 diabetes, sleep apnea, arthrosis of the hip or knee, polycystic ovary syndrome with an unmet wish for pregnancy, or treatment-resistant hypertension, or (2) BMI ≥ 40 kg/m^2^ with an obesity-related health risk other than those specified in 1.

#### Exclusion criteria

Patients were excluded if they had a history of medical disorders with known effects on bone metabolism (hypo- and hyperthyroidism are allowed if treated and stable) or metabolic bone disease (osteoporosis is allowed). Patients with previous treatment with anti-osteoporotic agents and current treatment with oral glucocorticoids or other drugs with effects on bone metabolism (inhaled and topic glucocorticoids, anti-epileptic agents, anticonceptives or other agents with estradiol are allowed) were excluded. Other exclusion criteria were pregnancy or breastfeeding, chronic kidney disease with estimated GFR < 45 ml/min, hypocalcemia, and hypersensitivity to bisphosphonates, mannitol, or sodium citrate. Fertile women that participate in this study must accept to use contraception with intrauterine devices or oral contraceptive pills with estradiol until 1 year after the administration of zoledronic acid/placebo. In addition, before the administration of the study drug, all fertile women will be screened for pregnancy using a urine human chorionic gonadotropin (HCG) test.

#### Drop out criteria

Participants will be excluded from the study due to safety issues including declining renal function, fulfillment of exclusion criterion, at own request or due to lack of compliance including failure to achieve a compulsory 8% weight loss prior to surgery. Also, patients will be excluded if surgery is not performed within 59 days from the administration of the study medicine or if the surgery was not performed as planned.

### Bariatric surgery

Routine bariatric surgery (RYGB or SG) will be performed within an interval of 30 days and 8 months after study inclusion. Endoscopic surgery will be performed by one of three skilled surgeons. The RYGB procedure includes a reduction of the gastric pouch to 20–30 mL, a 60-cm bilio-pancreatic limb, and a Roux limb of 150 cm. The SG procedure includes a gastric-volume reduction of 75 to 80% by resecting the stomach alongside a 30-French endoscope beginning 3 cm from the pylorus and ending at the angle of His.

### Supplements with calcium and vitamin D

According to Danish guidelines, all subjects will be advised supplements with calcium (citrate or carbonate) 400 mg two times daily and vitamin D 38 µg daily. Subjects will be advised to take supplements from inclusion and throughout the study. In case of serum 25-hydroxy-vitamin D levels below 25 nmol/l, a loading dose of 100,000 units vitamin D3 will be given orally.

### Sequence generation

After informed consent and baseline assessments, subjects will be randomized into two groups: zoledronic acid or placebo. The randomization sequence will be created using computer-generated software [28] stratifying patients with a 1:1 allocation using random block sizes of 2, 4, and 6. A randomization code stratifying an equal number of subjects having RYGB or SG into each study arm will be applied. Subjects will be blinded during the whole study. The investigator was responsible for performing randomization.

### Allocation concealment mechanism

After the acquisition of 12 months’ assessments, a non-member of the study team will prepare an un-blinded, anonymous data report for reporting of 12 months’ outcomes. All members of the study team remain blinded throughout the study.

### Intervention

Subjects will receive a single dose of zoledronic acid 5 mg or placebo 21 days before bariatric surgery (an interval of 59 to 7 days is accepted). Zoledronic acid or placebo will be administered in a solution containing 100-ml normal saline and slowly infused intravenous (≥ 15 min). Due to the risk of anaphylaxis, subjects are observed at least 30 min on the study site after the infusion. The Pharmacy, University hospital of Southern Denmark, will prepare the study medicine on the day of administration. Preparation is performed at a separate location in the hospital away from the research facility. Zoledronic acid or placebo solutions will be identical and labeled only with the randomization number of the particular subject.

### Endpoints

All outcomes will be assessed at baseline and 12 and 24 months after surgery. Table 1 shows a brief overview of primary and secondary endpoints in the study.

**Table 1:** Primary and secondary endpoints

	Measurement	Site	Time (months)
Primary endpoints			
vBMD	QCT	Lumbar spine (L1–L2)	12
Secondary Endpoints			
vBMD	QCT	Lumbar spine (L1–L2)	24
vBMD	QCT	Proximal femur	12 and 24
aBMD	DXA	Hip Lumbar spine	12 and 24
Lean and fat mass	DXA	Whole body	12 and 24
Bone microarchitecture	HR-pQCT	Distal radius Distal tibia	12 and 24
Cortical bone strength	Microindentation	Mid tibia	12 and 24
Biochemical markers of bone remodeling	CTX1 P1NP	Blood	12 and 24
Muscle strength	Dynamometer	Handgrip Knee extension/flexion Foot dorsi/plantar flexion Shoulder elevation	12 and 24
Physical function	SPPB T25FWT 2MWT Stair climb		12 and 24
Physical activity	IPAQ		12 and 24

Time is measured in months; *QCT* Quantitative computed tomography, *DXA* Whole-body dual-energy X-ray absorptiometry, *HR-pQCT* High-resolution peripheral quantitative computed tomography, *CTX-1* Carboxy-terminal type-1 collagen, *P1NP* Procollagen type-1 amino-terminal propeptide, *SPPB* Short Physical Performance Battery, *T25FWT* Timed 25-ft walk test, *2MWT* 2-min walk test, *IPAQ* The International Physical Activity Questionnaire

#### Primary endpoints

The primary endpoint is percentage change in volumetric BMD (vBMD) at the lumbar spine (L1–L2) measured by a non-contrast enhanced abdominal/pelvic quantitative computed tomography (QCT) (Siemens SOMATOM FORCE; Siemens Healthcare AG, Erlangen, Germany). The obtained images will be transferred to a QCT workstation and analyzed using Mindways QCT pro software (Mindways Software Inc., Austin, TX, USA). QCT is a multi-planar, three-dimensional bone densitometry imaging device and is chosen as the primary outcome since QCT provides detailed and accurate descriptions and analysis of bone shape, size, and architecture at macroscopic level, which is in contrast to DXA that only provides information about areal BMD (aBMD) [29].

#### Secondary endpoints

##### Anthropometrics and body composition

Fat and lean mass will be assessed by a whole-body dual-energy X-ray absorptiometry (DXA) scan (Hologic Horizon A; Waltham, MA, USA). DXA is a low resolution, uniplanar, two-dimensional bone densitometry imaging device that assesses mass quantities and densities for both full-body and segmental projections. DXA is chosen due to its ability to effectively differentiate hard tissue from soft tissue, and fat mass from lean mass.

Height and weight will be measured by wall-mounted stadiometer and weight scales (Seca 899, SECA GmbH, Hamburg, Germany). Waist circumference will be measured in the middle of the distance between the 12th rib and the anterior–superior iliac spine while keeping the measuring tape horizontally. Hip circumference will be measured across the widest part of the hips with the measuring tape horizontally.

##### Bone mass

Changes in wbBMC, aBMD of the hip (total hip region) and lumbar spine (L1-L4) will be measured by DXA.

##### Bone microarchitecture

Assessment of bone geometry, vBMD, cortical and trabecular microarchitecture, and estimated bone strength at the distal radius and tibia will be performed using high-resolution peripheral quantitative computed tomography (HR-pQCT) (XtremeCT II, Scanco Medical AG, Brutisellen, Switzerland). HR-pQCT provides information at the microscopic level about cortical and trabecular microarchitecture [29]. The reported outcomes will be compartmental geometry, vBMD, bone volume per trabecular volume (BV/TV), trabecular number, trabecular thickness, trabecular separation, cortical thickness, cortical porosity, and finite element analysis estimates of biomechanical competences. A detailed description of the scanning protocol and the image analyses have previously been published [30–32].

##### Cortical bone strength

Assessment of cortical Bone Material Strength index (BMSi) at the mid-tibia will be performed using impact microindentation (The OsteoProbe, Active Life Scientific, CA, USA). BMSi is a marker of bone stiffness. Microindentation is a minimally invasive procedure with minimal experienced pain and does not affect the ability of the individual to walk immediately afterwards. Briefly, the microindentation procedure consists of three steps: (1) subcutaneous local anesthesia of the mid-tibia region; (2) penetrating the skin and periosteum with the probe until it is reaching the bone cortex. A total of ten indentations are performed with the probe positioned perpendicular to the bone surface; (3) last, a single indentation in a reference material (the BMSi-100 Reference Material (polymethyl methacrylate)) is performed. BMSi is defined by the ratio of the penetration of the probe into the bone compared to a reference standard material and expressed in absolute units. A detailed description of the procedure is found elsewhere [33].

##### Biochemical calcium metabolic markers

Markers of bone formation (procollagen type-1 amino-terminal propeptide (P1NP)) and bone resorption (carboxy-terminal type-1 collagen (CTX-1)) as well as calcium, parathyroid hormone, and 25-hydroxy vitamin D will be measured using standardized equipment. Blood samples will be taken after an overnight fast and stored a − 70 °C in a biobank until analyses.

##### Muscle strength

Dynamic and isometric knee flexor/extensor (KF/KE) and ankle dorsi/plantar flexor (DF/PF) strength of the non-dominant leg will be measured in an isokinetic dynamometer (Biodex, System 4, Biodex Medical System Inc, New York, USA). The order of assessment will be dynamic contraction prior to isometric, and KF/KE prior to DF/PF. For the dynamic contractions, the subject will perform three attempts separated by 30-s rest in between. The angular velocities will be 180°/s and 90°/s for KF/KE and DF/PF respectively. For the isometric contractions, the subjects will perform three to five attempts lasting 3 s with 60-s rest in between. KF and KE will be performed in fixed positions of 30° and 75° of the knee angle and for DF and PF will be performed in 20° PF and 0° PF, respectively. For all dynamic and isometric attempts, the subjects will be instructed to relax, and after an auditory signal attempt to contract as “hard and fast” as possible. The reported outcomes will be muscle force normalized to body mass (Nm Kg^−1^).

Maximal shoulder elevation strength will be measured with Bofors MODEL dynamometer (Bofors MODEL dynamometer, Bofors Elektronic, Karlskoga, Sweden) mounted in a reproducible standardized setup [4]. The subjects perform 3–5 maximal isometric shoulder elevation in a seated position with 60-s rest in between.

Maximal handgrip strength (HGS) of the non-dominant hand will be measured by handgrip dynamometer (Jamar Plus, Patterson Medical, Warrenville, IL, USA). HGS measurements will be conducted in accordance with the recommendation from the American Society of Hand Therapists [34].

##### Physical function

Short Physical Performance Battery is a set of tests that determine mobility and function in some daily living activities, such as balance, walking speed, and strength. Briefly, SPPB has been validated to assess lower extremity function and balance in older adults [35]. SPPB includes three balance tests that increase in difficulty: parallel-, semi-tandem, and tandem stand. Walking speed is assessed by performing two trials of 3-m walks at the subject’s usual pace. Leg strength will be assessed by a chair stand test with five repeats performed as quickly as possible. SPPB will be scored according to guidelines [35].

In addition, a short walking test (T25FWT) will be used to assess the subject’s fast walking capacity and ability to initiate propulsive movement. Subjects will perform two 25-ft (7.6 m) walks as fast as possible from standing position. The fastest attempt will be selected for further analysis and is reported as velocity (m s^−1^) and time (s).

A 2-min walking test (2MWT) will be used to assess walking endurance capacity. The subject’s is instructed to walk as many rounds as possible on a 20-m track for 2 min. 2MWT will be reported as velocity (m s^−1^) and distance (m).

A 9-step stair (depth 28.5 cm, height 17 cm) ascend test will be used to assess the subject’s stair climbing abilities and functional power ((body weight × gravity (9.81 N kg^−1^) × stair height (1.53 m)/time). The subjects will be instructed to ascend 9-steps one step at a time as fast as possible. The subjects are allowed to use assistive devices and the stair railing if needed. Two attempts are performed, and the fastest attempt will be selected for further analysis and be reported as velocity (m s^−1^), time (s), and power (watt).

##### Physical activity

Changes in physical activity and exercise habits during the study may impact bone and muscle outcomes. To assess potential confounding, the physical activity and exercise habits will be assessed using the International Physical Activity Questionnaire (IPAQ). Physical activity will be assessed in at baseline, 12 and 24 months. The reported activities will be transformed to metabolic equivalent/week and the subjects are categorized as having a low, moderate, or high physical activity level according to guidelines [36]. IPAQ is chosen since Ekelund et al. [36] have shown that the short, last 7-day version of the IPAQ has acceptable validity for assessing MET/week in adults.

### Safety

Safety will be monitored in accordance with Good Clinical Practice Guidelines from the time of inclusion and until 12 months after surgery. All serious adverse events (SAE) and reactions (SAR) are reported to The Regional Committees on Health Research Ethics for Southern Denmark and The Danish Health and Medicines Authority. The GCP unit at Odense University Hospital will audit the study. At the 1- and 12-month visits, subjects will be specifically asked regarding adverse events. In addition, subjects are informed to notify the study site in case of adverse events or reactions to ensure continuous registration and reporting. Four weeks after surgery blood samples are collected to assess the occurrence of hypocalcemia and renal function. Fertile women that participate in the study accept to use intrauterine contraceptive devices or oral anticonception pills containing estrogen from inclusion and until 1 year after the administration of the study intervention. Screening for pregnancy using a urine HCG test is performed on the day of administration of the study intervention. Harms related to participation in the study are covered by the hospital’s insurance. All examinations performed in the study including QCT and DEXA scans and blood samples are assessed by a radiologist and/or an endocrinologist. In the case of random findings, the subjects will be contacted and offered additional examination and treatment if needed. In addition, advice on future clinical care related to musculoskeletal health is provided at study completion.

### Auditing, inspection, and monitoring

Direct access to source data is allowed for monitoring, auditing, and inspection of the study. The GCP unit at Odense University Hospital will be responsible for regular and notified monitoring of the study. Non-notified and notified auditing and inspection can be performed by The Danish Medicines Agency, The Regional Committees on Health Research Ethics for Southern Denmark or The Danish Data Protection Agency.

### Protocol amendments

Protocol amendments regarding trial design, data collection procedure, or aspects related to the safety or quality of the study drug are reported to The Regional Committees on Health Research Ethics for Southern Denmark and The Danish Medicine Agency. Other amendments that do not substantially impact the aspects listed above (non-substantial amendments) are only notified to the relevant authorities.

### Statistical analysis

#### Sample size determination

The sample size estimation is based on the primary outcome change in lumbar spine vBMD during12 months after surgery. A decline from 194 to 182 mg/cm^3^ is assumed (mean values with SD of 27 mg/cm^3^) [17] in those receiving placebo while those receiving zoledronic acid are assumed to have unchanged BMD. Then, a total of 21 subjects in each group will be sufficient to detect a statistically significant difference between the groups (repeated measures estimation, power 0.80, alpha 0.05, correlation 0.86). To allow for dropouts we aim to include 30 patients in each study arm. To ensure participant retention to the follow-up assessment, a study member will regularly be in contact with the participant by mail and phone throughout the whole study.

#### Statistical methods and data managing

Primary and secondary outcomes will be analyzed according to the intention to treat principle and subsequently per protocol. Missing data will be handled by multiple imputations, assuming missing values are randomly distributed. To assess the effects of the intervention, an age- and sex-adjusted mixed effect model with repeated measures including a term for the interaction of group (zoledronic acid or placebo) and time will be used to assess changes from baseline to 12 and 24 months after surgery.

Study data are stored using REDCap (Version 9.1.15 – © 2020 Vanderbilt University, TN, USA), an electronic data capture tool hosted at the University of Southern Denmark [37].

## Discussion

This is the first study to assess the effects of zoledronic acid on bone and muscle outcomes after bariatric surgery. In a 2-year randomized, double-blinded, placebo-controlled study effects of zoledronic acid on surrogates markers of bone strength including bone mass, architecture, and bone quality will be assessed. In addition, effects on muscle mass and strength and biomarkers of physical function are evaluated.

Although bariatric surgery has well-documented positive effects on diabetes and cardiovascular disease, it has become clear that adverse reactions occur in the muscular-skeletal system. Clinical guidelines advise supplements with calcium and vitamin D after bariatric surgery yet prospective studies with up to 7 years of follow-up have documented ongoing bone loss despite such recommendations. In addition to calcium and vitamin D, other non-pharmacological interventions have been suggested to prevent bone loss following bariatric surgery. For example, a 2-year randomized controlled trial found that a regimen of high-dose vitamin D, calcium, high protein intake, and a physical activity program was able to reduce but not prevent the negative impact on BMD and accelerated bone turnover [5]. Similar results were reported following combined supervised aerobic and resistance training [38]. After bariatric surgery, patients also experience a substantial decline in lean body mass assessed 2 years after surgery [5]. While the overall physical activity may be improved in parallel with the loss of weight, absolute muscle strength declines after surgery [4]. Gains in absolute muscle strength can be achieved following bariatric surgery with progressive moderate-intensity strength training with additional protein supplementation [15] or combined aerobic and strength training [38]. However, combined aerobic and strength training or strength training in combination with protein supplementation was insufficient to prevent muscle loss [15, 38]. This loss of muscle strength may cause a higher risk of falls, which in combination with the reduction in bone strength, contributes to the increase in fracture risk. Therefore, interventions that can secure muscular-skeletal health after bariatric surgery are warranted.

In postmenopausal osteoporosis, treatment with zoledronic acid reduces the risk of clinical vertebral fractures with approximately 75% and the fracture-reducing efficacy is generally well reflected in changes in bone mass during treatment [16]. With the number of subjects and short duration in the current study, a reduction in fracture occurrence is not expected; rather, the aim is to document effects on bone mass and other surrogates of bone strength to make probable that bisphosphonates are also effective for preserving bone health in a setting of bariatric surgery. When used in osteoporosis, zoledronic acid is administered once yearly; however, a fracture-reducing efficacy has also been observed with less frequent administration [39]. To assess if such longer-term effects are also seen in this setting, we chose to administer zoledronic acid once preoperatively while assessing outcomes after both 12 and 24 months.

The potential effects of bisphosphonates in muscle tissue may be attributed to the hormonal and mechanical interaction between the bones and muscles [24]. In a setting of bariatric surgery where large declines in lean body mass are observed, we aim to explore this proposed effect of bisphosphonates on muscle outcomes including muscle strength, mass, and physical function.

In general, obesity is related to high bone mass, likely a result of large mechanical demands on the skeleton given a high body weight. It has however been observed that bone strength in people with obesity may not fully adapt to body weight which may therefore lead to skeletal fragility [40, 41]. Accordingly, a number of studies have documented an increased risk of fractures at some, but not all, skeletal sites including proximal humerus, upper leg, and ankle fractures compared to normal weight individuals [42, 43]. Therefore, in obesity, bone mass may not reflect the risk of fracture to the same extent as in normal-weight individuals. For this reason, we chose not to use bone mass as a selection criterion but rather to include patients aged 35 years or older. At this age peak bone mass has been obtained, and therefore, we do not expect any further increments in bone mass during the study that could confound study findings.

We have chosen to include both RYGB and SG since both procedures induce similar reductions in bone mass in the short- [44–46] and intermediate-term [46] although some studies have shown some skeletal site-specific difference between the procedures [44]. Stratifying an equal number of subjects having RYGB or SG into each study arm will take account for such site-specific differences.

Since effects of osteoporosis medications are unclarified in this setting, results from this study, positive or negative, will be of importance for clinicians to offer evidence-based care. In case zoledronic acid antagonizes the bone metabolic imbalance induced by bariatric surgery, it will be of clinical relevance for both patients and clinicians. Should zoledronic acid enter clinical care in this setting, patients will easily be able to adhere to treatment given the administration once yearly. In addition, since the drug is no longer patented, health care costs will be low. Furthermore, this study will explore the potential effects of bisphosphonates in muscle tissue that will advance research in musculoskeletal pathophysiology and the proposed hormonal connections between muscle and bone.

### Limitations

There are some limitations of this study. First, bone or muscle biopsies are not obtained. Although effects of zoledronic acid are well documented in postmenopausal osteoporosis using bone biopsies, it would have been of interest to examine the potential effects of zoledronic acid in bone and skeletal muscle at the tissue and cellular levels in a setting of bariatric surgery. However, with the number of the examinations included in the study, we chose not to obtain biopsies for logistic reasons and since these are invasive procedures that can lead to complications. In case effects of zoledronic acid are observed in the muscle outcome,s this could be explored in a subsequent study. Second, bones and muscle are anabolic sensitive to mechanical loads such as physical activity and exercise [47]. Therefore, differences in physical activity and exercise habits would potentially compromise the interpretation of the outcomes. Accelerometers can accurately assess physical activity. However, accelerometers require technical expertise, specialized hardware, software, and individual programming [48]. Instead, IPAQ will be used to assess physical activity and exercise habits [36].

## Conclusion

This 2-year randomized, double-blinded, placebo-controlled study will assess the effects of zoledronic acid on surrogate markers of bone strength in people with severe obesity undergoing bariatric surgery. In addition, a potential effect of bisphosphonates in skeletal muscle is sought through assessments of muscle mass and strength and biomarkers of physical function.

## Dissemination policy

Results obtained after 1 year (including the primary study endpoint) will form the basis for a PhD thesis. Also, results will be presented at scientific meetings and submitted for publication in scientific journals with open access to ensure broad distribution. Also, we will communicate study findings to patient societies. All results, negative or positive, will be disseminated and reported in accordance with the CONSORT statement for trial reports.

## Trial registration

This trial was registered on 5 February 2021 at ClinicalTrials.gov, ID: NCT04742010. The first subject was recruited on 11 February 2021, and we expect to complete the recruitment of subjects within one and half year from the registration date (5–7-2022). The current protocol version is 1.1, date: 11–10-2019.

## Supplementary Information

**Additional file 1.** SPIRIT 2013 Checklist.

## Data Availability

The trial dataset is accessed by the investigators (SG and SGH). SG will perform the data analysis and takes responsibility for the integrity of the data. There are no results available from this study protocol. The public can access the data upon reasonable request.

## Abbreviations

2MWT: 2-Min walk test
aBMD: Areal bone mineral density
BMD: Bone mineral density
BMI: Body mass index
BMSi: Bone Material Strength index
BV/TV: Bone volume per trabecular volume
CTX-1: Carboxy-terminal type-1 collagen
DXA: Whole-body dual-energy X-ray absorptiometry
GIP: Gastric inhibitory protein
GLP 1: Glucagon-like-peptide 1
GLP 2: Glucagon-like-peptide 2
HR-pQCT: High-resolution peripheral quantitative computed tomography
IPAQ: International Physical Activity Questionnaire
P1NP: Procollagen type-1 amino-terminal propeptide
QCT: Quantitative computed tomography
RYGB: Roux-en-Y-gastric bypass
SAE: Serious adverse events
SAR: Serious adverse reactions
SG: Sleeve gastrectomy
SPIRIT: Standard Protocol Items Recommendations for Interventional Trials
SPPB: Short Physical Performance Battery
T25FWT: Timed 25-ft walk test
vBMD: Volumetric bone mineral density
